# Notes and News

**Published:** 1891-01-17

**Authors:** 


### NOTES AND NEWS.

COLLECTED AND COMMUNICATED.

MR. JONATHAN HUTCHINSON, F.R.S., lectured to a large and distinguished audience in the Examination Hall, Savoy Street, on Tuesday last. The subject of the lecture, which was the first of a course of eight post-graduate lectures, was "Lupus in General, with special reference to its connection with Tuberculosis." The lectures are peculiarly well-timed, and afford practitioners and students a unique opportunity of hearing an exposition of the mature views of one of the most laborious, experienced, and independent-minded surgeons in the world. We reserve comment on the lecture itself until next week. Among those present were Sir Andrew Clark, Sir Joseph Lister, and other gentlemen eminent in science and medicine.

THE next meeting of the Hospitals Association will be held on Wednesday, the 21st inst., at eight p.m., in the Board Room of Charing Cross Hospital. A paper will be read by Mr. L. S. Bristowe, barrister-at-law, on "The Legal Restrictions on Gifts to Charity." The offices of the Association have been moved to 140, Strand, and tickets can be had by applying to the Secretary there.

THE subscribers to the Coldstream Cottage Hospital held their second annual meeting on December 23rd. Mr. McLean, Hon. Secretary, read a most satisfactory report of the year's work. Sixty-three cases have been under treatment in the hospital, and nearly all were more or less improved before leaving, while twenty-two were entered on the register as "entirely cured." On application from the Coldstream Parochial Board the necessary steps were taken to alter a law of the Constitution, which excluded persons who were in receipt of parochial relief. The managers are now able to receive such cases if desired by the Board, and several such patients have already received successful treatment. Good work has been done by district nursing, and visits from the hospital nurses has been most gratefully received. The financial statement shows that the income for the year amounted to £328 0s. 11d., including a balance from last year of £57 13s. The expenditure was £285 5s. 8d., so the management have a balance of £42 15s. 3d.

THE annual ball in aid of the funds of Grantham Hospital took place on New Year's Day at the Guildhall, and passed off most successfully. The total attendance was close upon 200, and the ball is described as one of the most enjoyable and successful of the many memorable gatherings of the kind which have been organised on behalf of the hospital. On the following evening a dance was given in the same hall, the proceeds of which were to be divided between the Nurse Fund and the Self-aiding Dispensary, both excellent and deserving objects. There was a capital attendance, and the arrangements were all of a very satisfactory character.

SINCE it was founded ten years ago, Bolingbroke House Pay Hospital, has steadily increased in popularity, and it now only needs more private rooms to make it entirely self-supporting. That such a condition is most desirable, will be obvious to every one, and it is therefore to be hoped that the managers will find no difficulty in raising the £1,500, which is the estimated cost of the proposed wing. As this will contain not only more private rooms, but also quarters for nurses and servants, for whom at present an adjacent house is rented, it will add greatly to the economical working of the present house. The medical and surgical reports show that much has been done during the year for the relief of middle-class invalids, and the patients have expressed their gratitude in emphatic words, and one at least in deed as well, if an anonymous note of £50, sent with the simple words, "a thank offering," is to be ascribed to a benefited patient. Those who are not in a position to pay the full amount of their expenses, which average about two guineas weekly, are received on paying a reasonable proportion. The institution is in this respect a charity, the more deserving of support, in that it reaches the class most difficult to approach with help. Clerks, governesses, and professional men and women form a large proportion of the patients received, and to such the benefits of the institution are inestimable.

THE Manchester Infirmary Board met last week to consider the report of the special committee appointed in September to go into the question of the proposed extension of the institution. This committee was appointed on the representation of the Medical Board, who stated that the Infirmary was urgently in need of alterations and improvements. The committee were asked to consider, first, was there an undue pressure upon the accommodation provided for in-patients, and second, if there was, could that pressure be relieved by making any alteration in the existing system by which those patients were received? After an exhaustive inquiry, the committee had come to the conclusion that there was undoubted pressure on the demands for the admission of in-patients, and they strongly recommended the addition of 120 beds to the present Infirmary. They also urged that there should be increased provision for the purposes of operations, laboratories, and waiting-rooms. The committee proposed that the opinion of four architects be obtained as to the best way of carrying out these recommendations. The Chairman (Mr. E. S. Heywood) moved that the report be approved of and adopted, and the same committee be empowered to take the necessary steps for obtaining plans, &c., and to report to the Board thereon as early as possible. Mr. J. W. Maclure, M.P., seconded the motion, and said that they had no other course open to them except to extend the present buildings to a limited degree, and beyond that limit he hoped they would not go, as he was strongly in favour of keeping a large open space round the building. The scheme which was proposed would extend the benefits of a hospital, which he believed was second to none in the country. The motion was unanimously adopted.

THE following vacancies are announced this week, the amount of salary in each case being given after the name of the institution :—

**Resident Medical Officers.**

Hospital for Infectious Diseases, Newcastle-upon-Tyne, Medical Assistant—£50, board, &c.
Kent and Canterbury Hospital, Assistant House Surgeon and Dispenser—£50, board, &c.
Carlisle Dispensary, House Surgeon—£130, rooms.
Stockport Infirmary, Assistant House Surgeon—board, &c.
General Hospital, Birmingham, Assistant House Surgeon—board, &c.
Lancashire County Asylum, Pathological Assistant Medical Officer—£200, rooms, &c.
Cumberland Infirmary, Carlisle, House Surgeon—£70, board, &c.
Ingham Infirmary, Senior House Surgeon—£60, board, &c.
City Asylum, Birmingham, Clinical Assistant—board, &c.
Denbighshire Infirmary, House Surgeon—£85, board, &c.

**Non-Resident Medical Officers.**

County Borough of St. Helen's, Assistant Medical Officer—£200.
County of Dumfries, Medical Officer—£300.
Hambledon Union, Medical Officer—£60.
City Council of Ayrshire, County Medical Officer—£450.
Dental Hospital of London, Surgeons.

---

IN speaking the other week of the homes for children suffering from open wounds, &c., and long cases such as hip disease, mention should have been made of St. Monica's Home Hospital, Brondesbury, which was founded in 1874 for the reception of such cases. The last report shows that during 1889 forty-three patients were under treatment in this institution, not a single death occurred, while twelve patients left the Home, some of them in a position to earn their own livelihood, and some to continue their school course. A small sum is charged for the admission of each patient, and every effort is made to impart to them such knowledge as will help them to maintain themselves hereafter, and no pains are spared to make every girl an efficient needlewoman. The expenditure for 1889 was £1,613 18s. 8d., and the receipts £1,736 0s. 11d., leaving a balance of £122 2s. 3d. to carry over. Thirteen patients were taken for six weeks to Sussex for change of air, and four under specia circumstances to the seaside.

---

MANY months have elapsed since the buildings of the Derbyshire General Infirmary were found to be in a deplorable condition so far as the sanitary arrangements were concerned, and the greater portion of the hospital was declared unfit for the reception of patients. Temporary wooden wards were erected and have answered their purposes very well; nevertheless the general public have repeatedly inquired what is being done to replace the old hospital in an efficient position, or towards the erection of another institution. Sir William Evans has now published a letter answering these questions most satisfactorily. A Building Committee has been appointed to go into the whole question, with power to call in such professional advice as they might find necessary. The Committee were of opinion that their best course would be to visit many of the best known modern hospitals in the country, which they accordingly did, and have thus obtained much valuable information. The Committee have now appointed Messrs. Young and Hall to be the architects. These gentlemen are specialists in hospital construction, and as they are already conversant with the state of affairs at the Infirmary they will no doubt be able to devise a scheme for a hospital on the most approved and modern scientific principles. It may be assumed that the Committee will soon be able to lay some definite and complete scheme before the Governors of the institution. Sir William Evans fears that the sum required to complete this great work cannot be less than £50,000.

THE Toxteth Guardians have appointed Messrs. Ellison to be the architects of the proposed Workhouse Infirmary in Toxteth Park. The buildings are to be arranged so as to be capable of erection in sections, so as to suit present needs, to be added to as necessity may require. The buildings are to be simple in design, so as to keep the cost to the lowest possible limit, and are to consist of as many wards as the site will permit, together with an administrative block, nurses' home, and porters' lodges.

---

SOME very successful "tableaux vivants" have been given at the Imperial Hotel, Cork, in aid of the Hospital for Women and Children. The entertainment was the first of a series which the friends of the hospital have promoted, as funds are sorely needed and cannot be obtained entirely by voluntary contributions. The hospital has not always been well served by its entertainments. On some occasions there has been a loss where there ought to have been a gain. In this case, however, in spite of the necessary outlay, a substantial gain is anticipated, which will more than relieve the pressing wants of the institution.

---

THREE concerts have been held in Leicester for the benefit of the Children's Hospital and the Infirmary. The concerts were of a miscellaneous description, and took place in the Royal Opera House, which was kindly placed at the disposal of the honorary committee by Captain Winstanly. The second of the series, which was, perhaps, the most successful, was under the direction of the Hon. Mrs. Sterling and Mrs. Adrian Hope. The programme was long and attractive, and included, besides vocal and instrumental music, some clever recitations by the Misses Webling. It was confidently hoped that a cheque for possibly over £1,000, might be handed over to the treasurers of the above institution.

---

DR. KOCH'S cure is being extensively tried in the Dublin hospitals, and some remarkable cures have been effected in cases of lupus and tubercular diseases of the joints. A little girl who had suffered from strumous disease of the joints since infancy, and had many operations for the removal of the diseased glands, was placed under Dr. Koch's cure on December 8th. She has received five injections, beginning at two, and increasing to six milligrammes, and from being a miserable little wreck of humanity, she has grown quite bright and healthy-looking. She has greatly improved in weight, all the sinuses from the excised glands have healed up, and the glands that threatened suppuration have become small and harder, and some have quite disappeared. Many cases of phthisis have been placed under the lymph treatment, but the results so far are mostly of a negative character.

---

THE annual general meeting of contributors to the Royal Infirmary of Edinburgh was held in the Council Chambers, Edinburgh. Lord Provost Boyd presided. The report submitted by the managers stated that the total number of patients treated from October 1st, 1889, to the same date in 1890 was 8,695. Of these 3,648 had been dismissed cured, 3,121 relieved, and 685 on other grounds, while 634 had died. The ordinary income, which was £31,552 6s. 9d., showed an increase of £1,044 11s. 5d. The ordinary subscriptions showed the large increase of £735 9s. 10d., although the subscriptions from Edinburgh itself marked a decrease of £145 16s. 10d., probably due to accidental causes. The expenditure for last year was £37,945 15s., exceeding that of the previous year by £2,028 5s. 11d. The building debt has been reduced by £4,116, leaving still due £6,580. It is expected that the extraordinary expenditure of the ensuing year will be very large, owing to the erection of two operating theatres, and a new nurses' home.

